# *N*′-[(*E*)-2-Hy­droxy-5-meth­oxy­benzyl­idene]-2-meth­oxy­benzohydrazide

**DOI:** 10.1107/S1600536812042389

**Published:** 2012-11-03

**Authors:** M. Syukri. Baharudin, Muhammad Taha, Nor Hadiani Ismail, Syed Adnan Ali Shah, Sammer Yousuf

**Affiliations:** a Atta-ur-Rahman Institute for Natural Product Discovery, Universiti Teknologi MARA (UiTM), Puncak Alam Campus, 42300 Bandar Puncak Alam, Selangor D. E. Malaysia; b Faculty of Applied Science, Universiti Teknologi MARA (UiTM), 40450 Shah Alam, Malaysia; c Faculty of Pharmacy, Universiti Teknologi MARA, Puncak Alam, 42300, Selangor, Malaysia; d H. E. J. Research Institute of Chemistry, International Center for Chemical and Biological Sciences, University of Karachi, Karachi 75270, Pakistan

## Abstract

The mol­ecule of the title compound, C_16_H_16_N_2_O_4_, adopts an *E* conformation about the azomethine C=N double bond. The dihedral angle formed by the benzene rings is 18.88 (9)°. The mol­ecular conformation is stabilized by an intra­molecular O—H⋯N hydrogen bond, which forms an *S*(6) ring. In the crystal, the mol­ecules are linked into chains parallel to [001] by N—H⋯O hydrogen bonds. The chains are further connected into a three-dimensional network by π–π stacking inter­actions with centroid–centroid distances of 3.6538 (10) and 3.8995 (11) Å.

## Related literature

For the applications and biological activity of Schiff bases, see: Panneerselvam *et al.* (2009 ▶); Khan *et al.* (2009 ▶); Jarahpour *et al.* (2007 ▶); Pandeya *et al.* (1999 ▶). For related structures, see: Taha *et al.* (2012*a* ▶,*b* ▶); Lu *et al.* (2008 ▶).
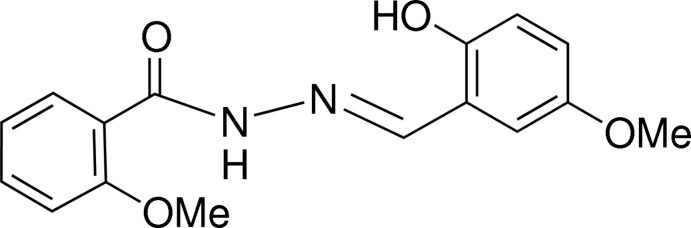


## Experimental

### 

#### Crystal data

C_16_H_16_N_2_O_4_
*M* *_r_* = 300.31
Monoclinic, 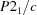

*a* = 14.5775 (13) Å
*b* = 11.0798 (11) Å
*c* = 9.5893 (9) Å
β = 99.872 (2)°
*V* = 1525.9 (2) Å^3^
*Z* = 4
Mo *K*α radiation
μ = 0.10 mm^−1^
*T* = 273 K
0.59 × 0.45 × 0.39 mm

#### Data collection

Bruker SMART APEX CCD area-detector diffractometer
Absorption correction: multi-scan (*SADABS*; Bruker, 2000 ▶) *T*_min_ = 0.946, *T*_max_ = 0.964
8886 measured reflections
2767 independent reflections
2210 reflections with *I* > 2σ(*I*)
*R*_int_ = 0.016

#### Refinement

*R*[*F* ^2^ > 2σ(*F* ^2^)] = 0.041
*wR*(*F* ^2^) = 0.124
*S* = 1.04
2767 reflections
208 parameters
H atoms treated by a mixture of independent and constrained refinement
Δρ_max_ = 0.15 e Å^−3^
Δρ_min_ = −0.15 e Å^−3^

### 

Data collection: *SMART* (Bruker, 2000 ▶); cell refinement: *SAINT* (Bruker, 2000 ▶); data reduction: *SAINT*; program(s) used to solve structure: *SHELXS97* (Sheldrick, 2008 ▶); program(s) used to refine structure: *SHELXL97* (Sheldrick, 2008 ▶); molecular graphics: *SHELXTL* (Sheldrick, 2008 ▶); software used to prepare material for publication: *SHELXTL*, *PARST* (Nardelli, 1995 ▶) and *PLATON* (Spek, 2009 ▶).

## Supplementary Material

Crystal structure: contains datablock(s) global, I. DOI: 10.1107/S1600536812042389/rz5014sup1.cif

Structure factors: contains datablock(s) I. DOI: 10.1107/S1600536812042389/rz5014Isup2.hkl

Supplementary material file. DOI: 10.1107/S1600536812042389/rz5014Isup3.cml

Additional supplementary materials:  crystallographic information; 3D view; checkCIF report

## Figures and Tables

**Table 1:** Hydrogen-bond geometry (Å, °)

*D*—H⋯*A*	*D*—H	H⋯*A*	*D*⋯*A*	*D*—H⋯*A*
O1—H1*A*⋯N1	0.83 (2)	1.87 (2)	2.605 (2)	146.0 (19)
N2—H2*A*⋯O3^i^	0.835 (17)	2.051 (17)	2.8258 (17)	154.2 (15)

Symmetry code: (i) 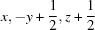.

**Fig. 1.**
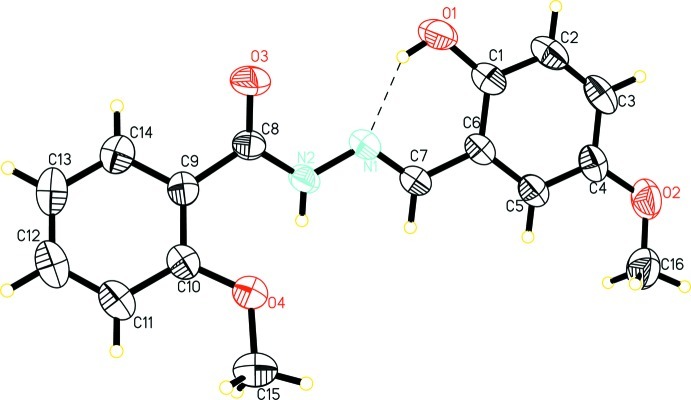
The molecular structure of the title compound with displacement ellipsoids drawn at the 30% probability level. The intramolecular hydrogen bond is shown as a dashed line.

**Fig. 2.**
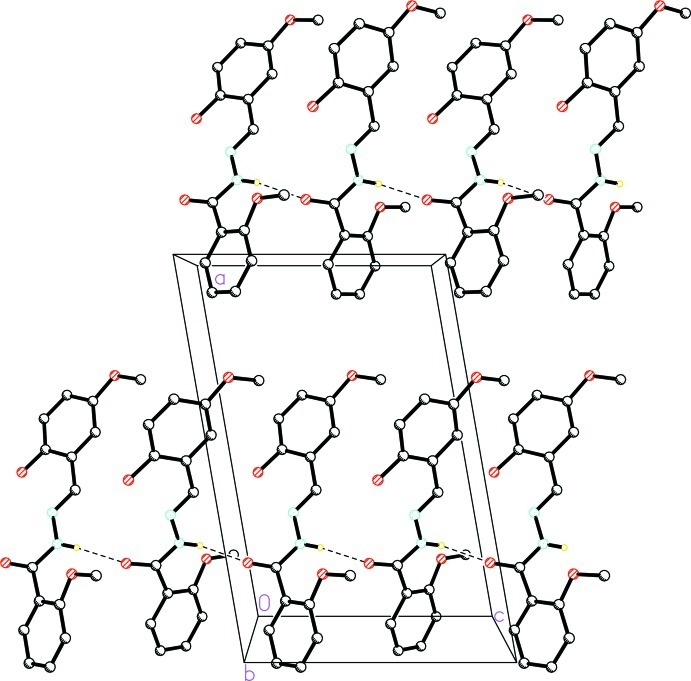
The crystal packing of the title compound. Only hydrogen atoms involved in hydrogen bonding (dashed lines) are shown.

## supplementary crystallographic information

### Comment

Applications of Schiff bases are reported in different fields of chemistry with a broad range of biological activities (Panneerselvam *et al.*, 2009, Khan *et al.*, 2009, Jarahpour *et al.*, 2007, Pandeya *et al.*, 1999). The title compound is a Schiff base synthesize as a part of our ongoing resaerch to study different biological activities of this medicinally important class of organic compounds.

The structure of title compound (Fig. 1) is similar to that of the previously published compound *N*'-(2-hydroxybenzylidene)-2-methoxybenzohydrazide monohydrate (Lu *et al.*, 2008), with the difference that the 2-hydroxy benzene ring is replaced by a 2-hydroxy-5-methoxy phenyl ring (C1–C6). The phenyl rings (C1–C6 and C9–C14) form an angle of 18.88 (9)°. Bond lengths and angles are similar to those observed in structurally related benzohydrazide derivatives (Taha *et al.*, 2012; Lu *et al.*, 2008). The *E* configuration of the azomethine olefinic bond is stabilized by an intramolecular O1—H1A···N1 hydrogen bond (Table 1) forming a ring of *S*(6) graph set motif. The crystal structure is stabilized by an intermolecular N2—H2A···O3 interaction forming chains running parallel to the [001] direction (Fig. 2). The chains are further linked into a three-dimensional network by π···π stacking interactions with centroid–centroid distances of 3.6538 (10) and 3.8995 (11) Å.

### Experimental

The title compound was synthesized by refluxing a mixture of 2-methoxybenzohydrazide (0.332 g, 2 mmol) and 2-hydroxy-5-methoxybenzaldehyde (0.304 g, 2 mmol) in methanol along with a catalytical amount of acetic acid for 3 hrs. The progress of reaction was monitored by TLC. After completion of reaction, the solvent was evaporated by vacuum to afford the crude product which was recrystallized by dissolving in methanol at room temperature to obtained needle-like crystals (0.504 g, 84% yield). All chemicals were purchased by Sigma Aldrich Germany.

### Refinement

H atoms on methyl, phenyl and methine carbon atoms were positioned geometrically with C—H = 0.96 Å (CH_3_) and 0.93 Å (CH), and constrained to ride on their parent atoms with *U*_iso_(H) = 1.5*U*_eq_(CH_3_) or 1.2*U*_eq_(CH). The H atoms on the nitrogen (N–H= 0.835 (17) Å) and oxygen (O–H= 0.84 (2) Å) atoms were located in a difference Fourier map and refined isotropically. A rotating group model was applied to the methyl groups.

### Crystal data

**Table:**

C_16_H_16_N_2_O_4_	*F*(000) = 632
*M**_r_* = 300.31	*D*_x_ = 1.307 Mg m^−^^3^
Monoclinic, *P*2_1_/*c*	Mo *K*α radiation, λ = 0.71073 Å
Hall symbol: -P 2ybc	Cell parameters from 3190 reflections
*a* = 14.5775 (13) Å	θ = 2.3–25.0°
*b* = 11.0798 (11) Å	µ = 0.10 mm^−^^1^
*c* = 9.5893 (9) Å	*T* = 273 K
β = 99.872 (2)°	Block, colourless
*V* = 1525.9 (2) Å^3^	0.59 × 0.45 × 0.39 mm
*Z* = 4	

### Data collection

**Table:**

Bruker SMART APEX CCD area-detector diffractometer	2767 independent reflections
Radiation source: fine-focus sealed tube	2210 reflections with *I* > 2σ(*I*)
Graphite monochromator	*R*_int_ = 0.016
ω scan	θ_max_ = 25.5°, θ_min_ = 1.4°
Absorption correction: multi-scan (*SADABS*; Bruker, 2000)	*h* = −17→16
*T*_min_ = 0.946, *T*_max_ = 0.964	*k* = −13→13
8886 measured reflections	*l* = −9→11

### Refinement

**Table:**

Refinement on *F*^2^	Primary atom site location: structure-invariant direct methods
Least-squares matrix: full	Secondary atom site location: difference Fourier map
*R*[*F*^2^ > 2σ(*F*^2^)] = 0.041	Hydrogen site location: inferred from neighbouring sites
*wR*(*F*^2^) = 0.124	H atoms treated by a mixture of independent and constrained refinement
*S* = 1.04	*w* = 1/[σ^2^(*F*_o_^2^) + (0.0648*P*)^2^ + 0.1784*P*] where *P* = (*F*_o_^2^ + 2*F*_c_^2^)/3
2767 reflections	(Δ/σ)_max_ < 0.001
208 parameters	Δρ_max_ = 0.15 e Å^−^^3^
0 restraints	Δρ_min_ = −0.15 e Å^−^^3^

### Special details

**Table:**

Geometry. All e.s.d.'s (except the e.s.d. in the dihedral angle between two l.s. planes) are estimated using the full covariance matrix. The cell e.s.d.'s are taken into account individually in the estimation of e.s.d.'s in distances, angles and torsion angles; correlations between e.s.d.'s in cell parameters are only used when they are defined by crystal symmetry. An approximate (isotropic) treatment of cell e.s.d.'s is used for estimating e.s.d.'s involving l.s. planes.
Refinement. Refinement of *F*^2^ against ALL reflections. The weighted *R*-factor *wR* and goodness of fit *S* are based on *F*^2^, conventional *R*-factors *R* are based on *F*, with *F* set to zero for negative *F*^2^. The threshold expression of *F*^2^ > σ(*F*^2^) is used only for calculating *R*-factors(gt) *etc*. and is not relevant to the choice of reflections for refinement. *R*-factors based on *F*^2^ are statistically about twice as large as those based on *F*, and *R*- factors based on ALL data will be even larger.

### Fractional atomic coordinates and isotropic or equivalent isotropic displacement parameters (Å^2^)

**Table:**

	*x*	*y*	*z*	*U*_iso_*/*U*_eq_	
O1	0.41411 (11)	0.06306 (13)	0.11043 (13)	0.0918 (4)	
O2	0.68838 (9)	0.10138 (14)	0.58074 (17)	0.1009 (4)	
O3	0.17476 (9)	0.24072 (14)	0.01591 (12)	0.0957 (5)	
O4	0.16599 (8)	0.47356 (10)	0.32496 (12)	0.0767 (3)	
N1	0.31001 (9)	0.20202 (11)	0.23724 (13)	0.0618 (3)	
N2	0.22889 (9)	0.26092 (12)	0.24670 (14)	0.0634 (4)	
C1	0.47885 (12)	0.07530 (15)	0.22962 (18)	0.0714 (5)	
C2	0.56411 (15)	0.01986 (17)	0.2354 (2)	0.0881 (6)	
H2B	0.5758	−0.0250	0.1584	0.106*	
C3	0.63154 (14)	0.03008 (17)	0.3527 (2)	0.0884 (6)	
H3A	0.6885	−0.0083	0.3548	0.106*	
C4	0.61614 (11)	0.09696 (16)	0.4685 (2)	0.0752 (5)	
C5	0.53108 (11)	0.15170 (14)	0.46503 (19)	0.0692 (4)	
H5A	0.5198	0.1959	0.5428	0.083*	
C6	0.46152 (10)	0.14167 (13)	0.34613 (16)	0.0612 (4)	
C7	0.37267 (11)	0.19998 (13)	0.34759 (17)	0.0629 (4)	
H7A	0.3612	0.2364	0.4303	0.076*	
C8	0.16545 (10)	0.28010 (13)	0.13077 (15)	0.0585 (4)	
C9	0.08121 (10)	0.34987 (13)	0.15057 (14)	0.0578 (4)	
C10	0.08163 (11)	0.44261 (14)	0.24890 (16)	0.0631 (4)	
C11	−0.00098 (14)	0.50192 (17)	0.2600 (2)	0.0822 (5)	
H11A	−0.0012	0.5626	0.3270	0.099*	
C12	−0.08169 (14)	0.47113 (19)	0.1728 (3)	0.0922 (6)	
H12A	−0.1367	0.5106	0.1818	0.111*	
C13	−0.08304 (12)	0.3835 (2)	0.0728 (2)	0.0883 (6)	
H13A	−0.1383	0.3641	0.0131	0.106*	
C14	−0.00128 (12)	0.32362 (16)	0.06098 (18)	0.0743 (5)	
H14A	−0.0019	0.2648	−0.0083	0.089*	
C15	0.17013 (18)	0.5590 (2)	0.4353 (3)	0.1167 (8)	
H15A	0.2339	0.5716	0.4783	0.175*	
H15B	0.1357	0.5295	0.5050	0.175*	
H15C	0.1437	0.6339	0.3974	0.175*	
C16	0.67844 (15)	0.1759 (2)	0.6962 (3)	0.1081 (7)	
H16A	0.7359	0.1775	0.7622	0.162*	
H16B	0.6299	0.1448	0.7421	0.162*	
H16C	0.6628	0.2563	0.6629	0.162*	
H2A	0.2202 (11)	0.2826 (14)	0.3268 (18)	0.066 (5)*	
H1A	0.3662 (16)	0.101 (2)	0.120 (2)	0.106 (7)*	

### Atomic displacement parameters (Å^2^)

**Table:**

	*U*^11^	*U*^22^	*U*^33^	*U*^12^	*U*^13^	*U*^23^
O1	0.1031 (10)	0.1066 (10)	0.0695 (8)	0.0320 (8)	0.0257 (7)	−0.0085 (7)
O2	0.0627 (8)	0.1096 (10)	0.1274 (11)	0.0180 (7)	0.0077 (8)	−0.0032 (9)
O3	0.1036 (10)	0.1328 (11)	0.0532 (7)	0.0220 (8)	0.0202 (6)	−0.0145 (7)
O4	0.0744 (8)	0.0750 (7)	0.0801 (7)	0.0077 (6)	0.0118 (6)	−0.0138 (6)
N1	0.0644 (8)	0.0624 (7)	0.0623 (8)	0.0105 (6)	0.0214 (7)	0.0013 (6)
N2	0.0642 (8)	0.0780 (8)	0.0509 (7)	0.0163 (6)	0.0180 (6)	−0.0018 (6)
C1	0.0783 (11)	0.0701 (10)	0.0719 (10)	0.0150 (8)	0.0298 (9)	0.0068 (8)
C2	0.0933 (14)	0.0870 (12)	0.0924 (13)	0.0288 (11)	0.0400 (12)	−0.0002 (10)
C3	0.0740 (12)	0.0836 (12)	0.1166 (16)	0.0274 (10)	0.0418 (12)	0.0122 (11)
C4	0.0571 (10)	0.0719 (10)	0.0989 (12)	0.0093 (8)	0.0204 (9)	0.0092 (9)
C5	0.0634 (10)	0.0658 (9)	0.0826 (11)	0.0059 (7)	0.0241 (9)	−0.0007 (8)
C6	0.0608 (9)	0.0569 (8)	0.0708 (9)	0.0071 (7)	0.0247 (8)	0.0039 (7)
C7	0.0648 (10)	0.0622 (8)	0.0654 (9)	0.0073 (7)	0.0211 (8)	−0.0030 (7)
C8	0.0666 (9)	0.0629 (8)	0.0488 (8)	−0.0017 (7)	0.0180 (7)	0.0036 (6)
C9	0.0581 (9)	0.0632 (8)	0.0539 (8)	0.0000 (7)	0.0142 (7)	0.0152 (6)
C10	0.0643 (10)	0.0622 (8)	0.0654 (9)	0.0052 (7)	0.0183 (8)	0.0104 (7)
C11	0.0774 (12)	0.0749 (11)	0.0991 (13)	0.0142 (9)	0.0293 (10)	0.0068 (9)
C12	0.0663 (12)	0.0863 (13)	0.1271 (17)	0.0149 (10)	0.0258 (12)	0.0237 (12)
C13	0.0587 (11)	0.0930 (13)	0.1090 (15)	−0.0021 (9)	0.0026 (10)	0.0306 (12)
C14	0.0737 (11)	0.0744 (10)	0.0733 (10)	−0.0056 (8)	0.0082 (9)	0.0145 (8)
C15	0.1156 (18)	0.1105 (17)	0.1212 (18)	0.0065 (13)	0.0127 (14)	−0.0508 (14)
C16	0.0801 (14)	0.1211 (18)	0.1162 (17)	0.0058 (12)	−0.0030 (12)	−0.0012 (15)

### Geometric parameters (Å, º)

**Table:**

O1—C1	1.359 (2)	C6—C7	1.450 (2)
O1—H1A	0.84 (2)	C7—H7A	0.9300
O2—C4	1.372 (2)	C8—C9	1.491 (2)
O2—C16	1.408 (3)	C9—C14	1.384 (2)
O3—C8	1.2138 (17)	C9—C10	1.394 (2)
O4—C10	1.362 (2)	C10—C11	1.392 (2)
O4—C15	1.413 (2)	C11—C12	1.365 (3)
N1—C7	1.2740 (18)	C11—H11A	0.9300
N1—N2	1.3671 (17)	C12—C13	1.362 (3)
N2—C8	1.3356 (19)	C12—H12A	0.9300
N2—H2A	0.835 (17)	C13—C14	1.386 (3)
C1—C2	1.379 (2)	C13—H13A	0.9300
C1—C6	1.396 (2)	C14—H14A	0.9300
C2—C3	1.366 (3)	C15—H15A	0.9600
C2—H2B	0.9300	C15—H15B	0.9600
C3—C4	1.385 (3)	C15—H15C	0.9600
C3—H3A	0.9300	C16—H16A	0.9600
C4—C5	1.376 (2)	C16—H16B	0.9600
C5—C6	1.394 (2)	C16—H16C	0.9600
C5—H5A	0.9300		
			
C1—O1—H1A	109.1 (15)	C14—C9—C10	118.52 (15)
C4—O2—C16	117.98 (14)	C14—C9—C8	117.34 (14)
C10—O4—C15	119.23 (15)	C10—C9—C8	124.08 (14)
C7—N1—N2	117.29 (12)	O4—C10—C11	123.63 (16)
C8—N2—N1	120.27 (12)	O4—C10—C9	116.54 (13)
C8—N2—H2A	121.8 (11)	C11—C10—C9	119.75 (16)
N1—N2—H2A	117.9 (11)	C12—C11—C10	120.09 (19)
O1—C1—C2	118.72 (16)	C12—C11—H11A	120.0
O1—C1—C6	122.06 (14)	C10—C11—H11A	120.0
C2—C1—C6	119.22 (17)	C13—C12—C11	121.09 (18)
C3—C2—C1	120.82 (17)	C13—C12—H12A	119.5
C3—C2—H2B	119.6	C11—C12—H12A	119.5
C1—C2—H2B	119.6	C12—C13—C14	119.35 (18)
C2—C3—C4	120.80 (16)	C12—C13—H13A	120.3
C2—C3—H3A	119.6	C14—C13—H13A	120.3
C4—C3—H3A	119.6	C9—C14—C13	121.11 (18)
O2—C4—C5	124.81 (17)	C9—C14—H14A	119.4
O2—C4—C3	116.11 (16)	C13—C14—H14A	119.4
C5—C4—C3	119.07 (18)	O4—C15—H15A	109.5
C4—C5—C6	120.71 (16)	O4—C15—H15B	109.5
C4—C5—H5A	119.6	H15A—C15—H15B	109.5
C6—C5—H5A	119.6	O4—C15—H15C	109.5
C5—C6—C1	119.36 (14)	H15A—C15—H15C	109.5
C5—C6—C7	118.83 (14)	H15B—C15—H15C	109.5
C1—C6—C7	121.80 (15)	O2—C16—H16A	109.5
N1—C7—C6	120.96 (14)	O2—C16—H16B	109.5
N1—C7—H7A	119.5	H16A—C16—H16B	109.5
C6—C7—H7A	119.5	O2—C16—H16C	109.5
O3—C8—N2	121.93 (14)	H16A—C16—H16C	109.5
O3—C8—C9	121.66 (14)	H16B—C16—H16C	109.5
N2—C8—C9	116.39 (12)		
			
C7—N1—N2—C8	171.24 (14)	N1—N2—C8—O3	4.2 (2)
O1—C1—C2—C3	179.69 (17)	N1—N2—C8—C9	−177.33 (12)
C6—C1—C2—C3	−0.6 (3)	O3—C8—C9—C14	29.3 (2)
C1—C2—C3—C4	−0.4 (3)	N2—C8—C9—C14	−149.17 (14)
C16—O2—C4—C5	−6.0 (3)	O3—C8—C9—C10	−147.80 (16)
C16—O2—C4—C3	175.16 (18)	N2—C8—C9—C10	33.75 (19)
C2—C3—C4—O2	180.00 (17)	C15—O4—C10—C11	9.0 (2)
C2—C3—C4—C5	1.1 (3)	C15—O4—C10—C9	−174.19 (17)
O2—C4—C5—C6	−179.68 (15)	C14—C9—C10—O4	−173.55 (13)
C3—C4—C5—C6	−0.9 (3)	C8—C9—C10—O4	3.5 (2)
C4—C5—C6—C1	0.0 (2)	C14—C9—C10—C11	3.4 (2)
C4—C5—C6—C7	179.48 (14)	C8—C9—C10—C11	−179.56 (14)
O1—C1—C6—C5	−179.50 (15)	O4—C10—C11—C12	175.27 (16)
C2—C1—C6—C5	0.8 (2)	C9—C10—C11—C12	−1.4 (3)
O1—C1—C6—C7	1.0 (2)	C10—C11—C12—C13	−0.8 (3)
C2—C1—C6—C7	−178.75 (15)	C11—C12—C13—C14	0.9 (3)
N2—N1—C7—C6	−178.73 (13)	C10—C9—C14—C13	−3.3 (2)
C5—C6—C7—N1	173.93 (14)	C8—C9—C14—C13	179.48 (14)
C1—C6—C7—N1	−6.6 (2)	C12—C13—C14—C9	1.1 (3)

### Hydrogen-bond geometry (Å, º)

**Table:**

*D*—H···*A*	*D*—H	H···*A*	*D*···*A*	*D*—H···*A*
O1—H1*A*···N1	0.83 (2)	1.87 (2)	2.605 (2)	146.0 (19)
N2—H2*A*···O3^i^	0.835 (17)	2.051 (17)	2.8258 (17)	154.2 (15)

Symmetry code: (i) *x*, −*y*+1/2, *z*+1/2.
